# Supplementary material for: Unexpected large impact of small charges on surface frictions with similar wetting properties
Source: Commun Chem. 2020 Feb 27;3:27. doi: 10.1038/s42004-020-0271-8 (PMC9814279; doi:10.1038/s42004-020-0271-8)
Supplement: Supplementary file 1 — Supplementary Information [file 42004_2020_271_MOESM1_ESM.pdf]

Supporting Information for *Unexpected Large Impact of Small Charges on Surface Frictions with Similar Wetting Properties* by Chunlei Wang et al.

Supplementary Note 1. Friction coefficient *λ* obtained from the correlation of friction forces versus time *t* on the *hexagonal* lattice structure surfaces

.Supplementary Figure 1 shows the comparison between the Green−Kubo estimate of the friction coefficient of liquid water on hexagonal lattice surfaces for various charge q. We have calculated the friction coefficient λ given by the plateau value at times interval of 1 ps. There is an evident increase in the friction coefficient as the charge q increases.

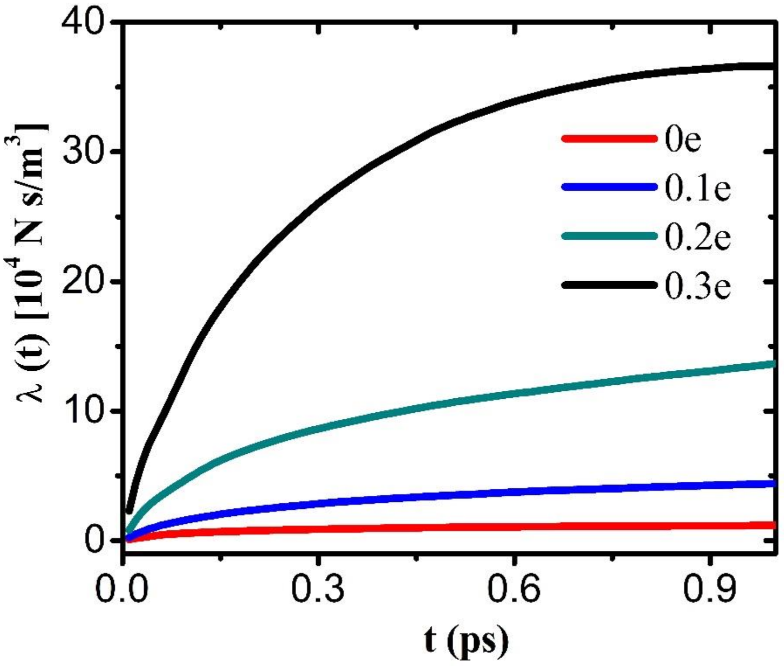

Supplementary Figure 1. Friction coefficient *λ* obtained from the correlation of friction forces versus time *t* on the hexagonal lattice structure surfaces.

Supplementary Note 2. Water density near the solid surfaces in z axis of various solid surfaces

We have also investigated the distribution of the water molecules near the solid

surfaces, where surface water density of the surface water shown in Supplementary Figure 2. Several density profiles on surface with different charge values overlap for most of the film height. The first peak, at a height of about 3.0 Å, hits a density maximum of ≈ 2.7 g/cm^3. It is shown that the density near the solid surfaces are almost identical, which is even more insensitive to discriminate the surface with different charges (see Supplementary Figure 2), even though the maximum variation of density values of water in the first peak can reach 9% when comparing q = 0 e with q = 0.3 e. These density results are also clearly inconsistent with the variation law of the friction coefficient in respect of the charge q.

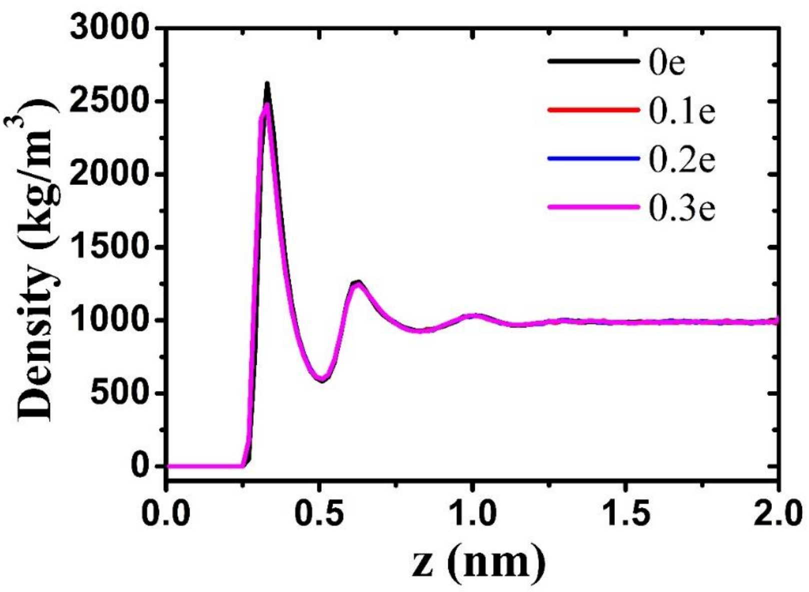

Supplementary Figure 2. Water density profiles for various charge values.

Supplementary Note 3. Probability distribution profile for the microscopic interaction energy E_{micro} of the hexagonal lattice surfaces.

We divide the surface area by small squares with area 0.25 Å^2. We calculated the average interaction E_{micro} of each water molecule of the first contact layer with the thickness of 0.5 nm locating in this area and all the solid surface atoms, and plot the energy profile. We find that the in-plane potential energy E_{micro} falls the range from -4.2 kJ/mol to -7.0 kJ/mol. We have plotted the probability distribution profile of

mircoscopic interaction energy E_{micro} for the surfaces with various charge q. Clearly, the energy E_{micro} probability profiles exhibits prominently more broadly distribution when charge q = 0 e increase to 0.3 e. This distribution is quite sharply in contrast to the insignificantly change of average interactions E between surface and the water molecules and the contact angles for various charge q values shown in Fig 2a in the main text.

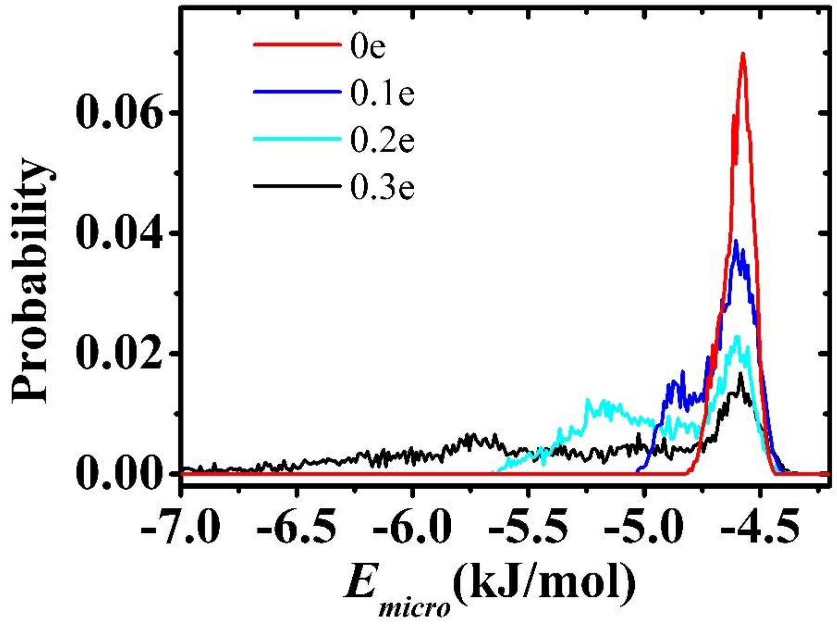

Supplementary Figure 3. Probability distribution profile for the microscopic interaction energy E_{micro} surfaces with various charge q.

Supplementary Note 4. Contact angles and the estimate of the friction coefficient of liquid water on *square* lattice solid surface in respect of the charge q

As any dissipation coefficient, the friction coefficient can be expressed via linear response theory in terms of a Green-Kubo (GK) relationship, relating λ to a correlation function of a fluctuating microscopic variable at equilibrium

$$\lambda = \frac{1}{Ak_B T}\int_0^\infty dt \langle F(t)F(0)\rangle_{equ} \qquad (1),$$

where $F(t)$ is the total tangential force acting along the x direction on the surface with area A and the average runs over equilibrium configurations, k_B is the Boltzmann

constant and T is the temperature. Using this equation, we have computed the friction coefficient λ at the solid/water interface in respect to the charge q. We have found that the surface charge values significantly affect the friction coefficient values, inconsistent with the contact angle values. Despite the same contact angles of around 56°±2°, the friction coefficient are 1.04×10^6 Ns/m^3 for q = 0.2 e and 1.2×10^3 Ns/m^3 for q = 0 e, where the former about 1000 times of magnitude larger than the latter. This clearly show that the relationship between the hydrophobicity characterized by contact angle and surface friction are deviating much on a very weakly polar surfaces. As q further increases to 0.3 e, the friction coefficient increase to 2.5×10^6 Ns/m^3 , which is more than 2000 times larger than that at q = 0 e, and the contact angle values decreases by 17% to the values of 45°±2°.

a

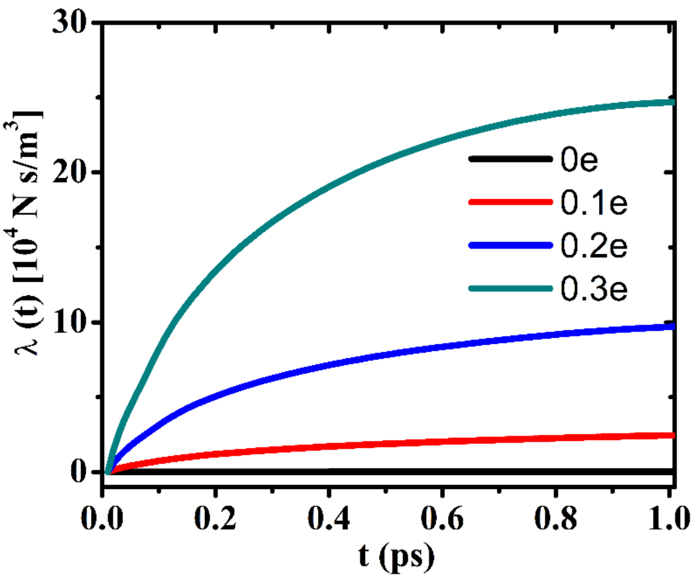

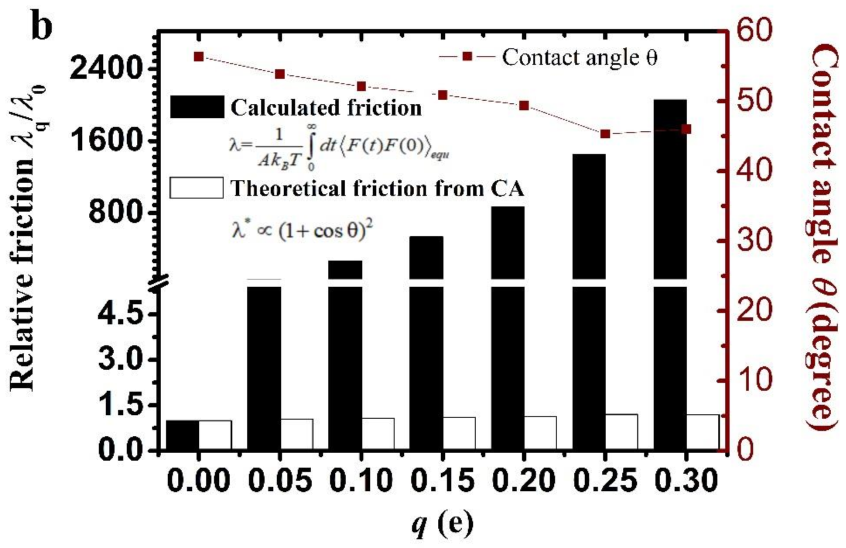

Supplementary Figure 4. (a) Friction coefficient λ obtained from the correlation of friction forces versus time t on the square lattice structure surfaces. (b) Contact angles and the estimate of the friction coefficient of liquid water on square lattice solid surface in respect of the charge q values from 0 e to 0.3 e.

We find that the in-plane potential energy E_{micro} distribution profiles become localized despite the small energy values of -6.4 kJ/mol to -9.0 kJ/mol on the square lattice surfaces, as the charge q increases from 0 e to 0.3 e. As shown in Supplementary Figure 5, as q = 0 e, there are many continuous area for the energy distribution profiles, which means smooth energy profiles without energy barrier that obstruct the water molecules to move. When the surface charge q further increases to 0.2 e, the color area becomes discontinuous and is separated by the large and small energy values.

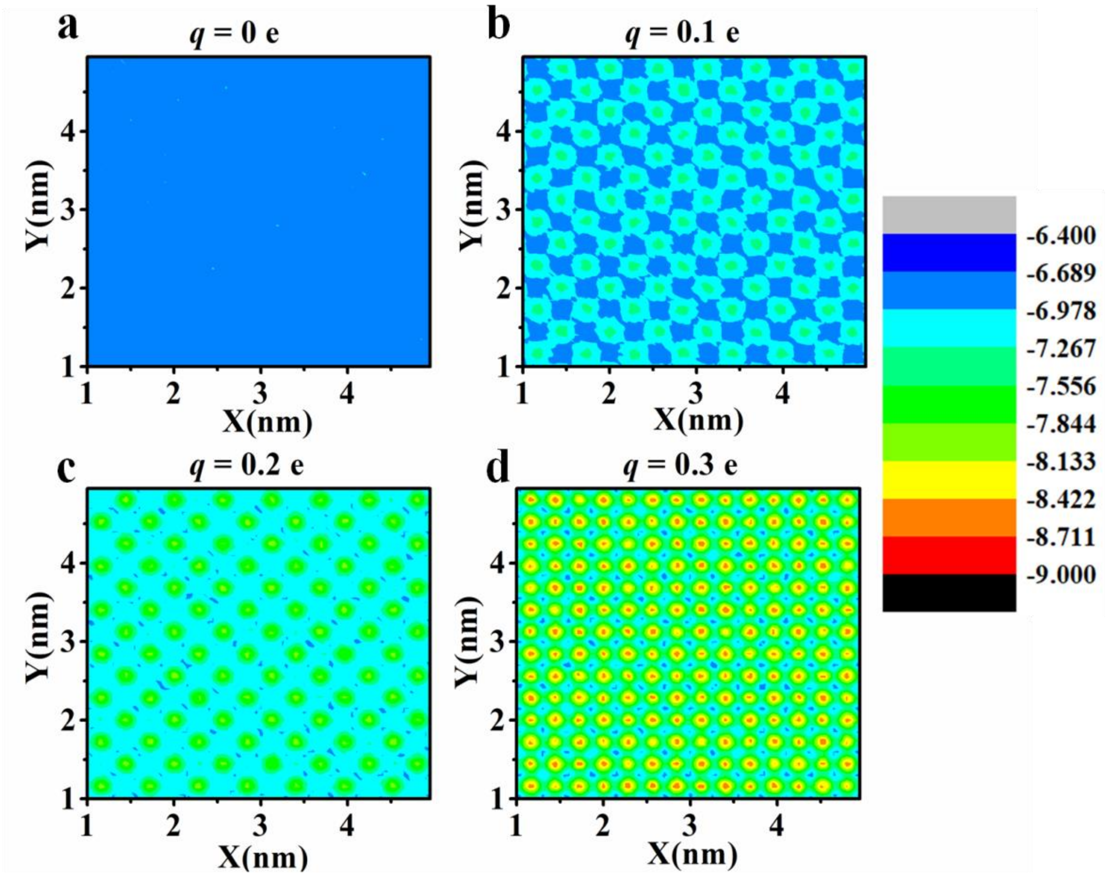

Supplementary Figure 5. Microscopic potential energy profiles of square lattice surfaces for different charge value *q* = 0 e (a), 0.1 e (b), 0.2 e (c) and 0.3 e (d), where the potential energy profiles is calculated for each water molecule in the first water layer experiences within the area 0.25 Å^2.

Supplementary Note 5 Dependence of microscopic energy variance (ΔE_{micro})^2, surface friction coefficient and the analysis of total interactions between surface-water on the surface charge *q*.

Based on the Green-Kubo relationship, the surface friction λ is proportional to the square of surface-water total tangential force $<F>^2$ [1]. For the various polar solid surfaces we study, the main difference lies in the surface charge q, which thus uniquely determine the difference of the total tangential force $<F_{xy}>^2$, different from the previous work that on the homogeneous Lennard-Jones atomic surfaces.[1] Due to the inhomogeneous charge distribution, the force $<F_{xy}>$ is roughly proportional to the potential corrugation ΔE_{micro}, thus $\lambda \sim <F_{xy}>^2$ $(\Delta E_{micro})^2 \sim q^2$. Thus, as charge value q

increases on these surfaces, the local potential corrugation increased, which is the main cause for the increase of the friction coefficient. [2,3] As shown in Supplementary Figure 6, the relative friction defined as λ_q/λ_0 and relative square energy $(\Delta E_{micro})_q^2/(\Delta E_{micro})_0^2$ are synchronously quadratically dependent of the charge q, further confirming the previous analysis.

The contact angles are significantly dependent of the total solid-water interactions, including the van der Waals E_{vdw} and electrostatic interactions E_{eletro}, as shown in Supplementary Figure 7. When the polarity q is smaller than 0.2 e, the van der Waals interactions are dominant, which are much smaller (<10%) than the electrostatic interactions.

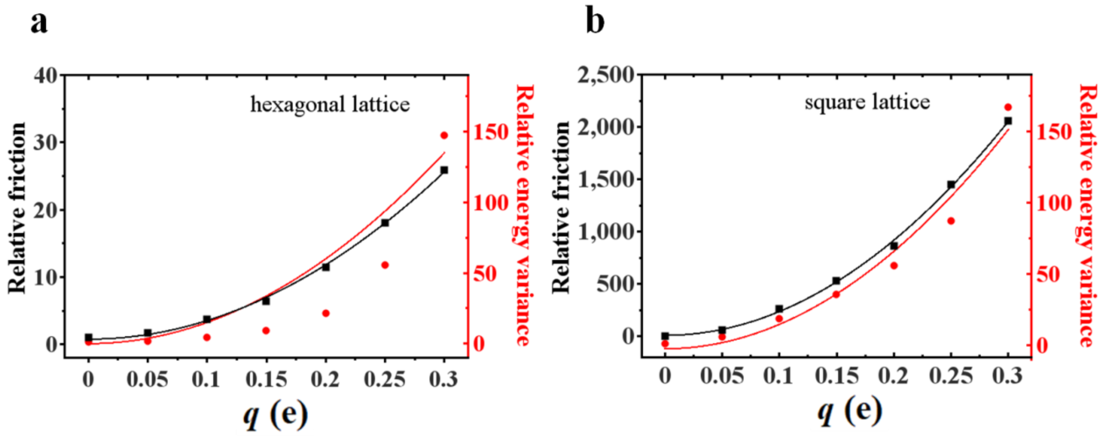

Supplementary Figure 6 Relative surface friction coefficient calculated based on the Green-Kubo relationship and relative rate of microscopic energy variance $(\Delta E_{micro})^2$, versus charge q for (a) hexagonal lattice structures and (b) square lattice structures. The black and red lines show the quadratically fit between the friction coefficient and variance $(\Delta E_{micro})^2$ dependent of the charge q.

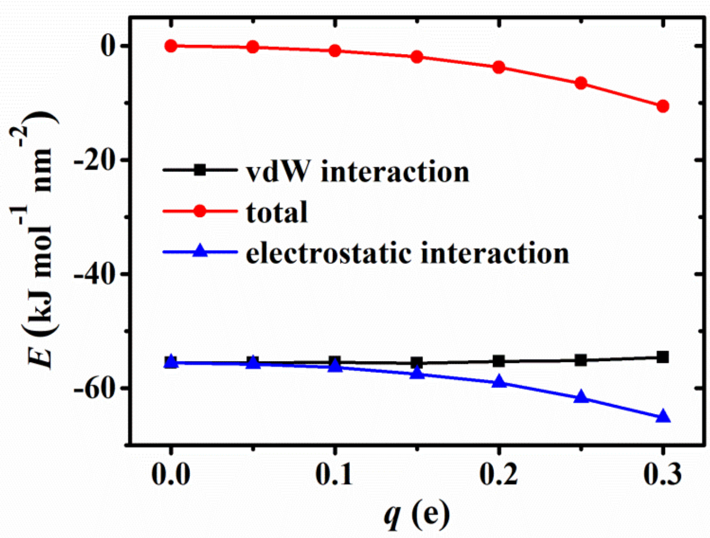

Supplementary Figure 7 Average interactions *E* between surface and the water molecules, the van der Waals and electrostatic interactions for various charge *q* values.

Supplementary References

1 Huang, D. M., Sendner, C., Horinek, D., Netz, R. R. & Bocquet, L. Water Slippage versus Contact Angle: A Quasiuniversal Relationship. *Phys. Rev. Lett.* **101**, 226101 (2008).

2 Tocci, G., Joly, L. & Michaelides, A. Friction of Water on Graphene and Hexagonal Boron Nitride from Ab Initio Methods: Very Different Slippage Despite Very Similar Interface Structures. *Nano Lett.* **14**, 6872-6877, (2014).

3 Bocquet, L. & Charlaix, E. Nanofluidics, from bulk to interfaces. *Chem. Soc. Rev.* **39**, 1073-1095 (2010).
